# Are there disparities in different domains of physical activity between school-aged migrant and non-migrant children and adolescents? Insights from Germany

**DOI:** 10.1371/journal.pone.0214022

**Published:** 2019-03-18

**Authors:** Anne K. Reimers, Patrick Brzoska, Claudia Niessner, Steffen C. E. Schmidt, Annette Worth, Alexander Woll

**Affiliations:** 1 Institute of Human Movement Science and Health, Faculty of Behavioral and Social Sciences, Technical University of Chemnitz, Chemnitz, Germany; 2 Health Services Research Unit, Faculty of Health/School of Medicine, Witten/Herdecke University, Witten/Herdecke, Germany; 3 Institute of Sport and Sport Science, Karlsruhe Institute of Technology, Karlsruhe, Germany; 4 Institute for Movement and Sports, University of Education Karlsruhe, Karlsruhe, Germany; University of Campania, ITALY

## Abstract

**Background:**

Large proportions of the populations in many European countries, including Germany, are migrants. Migrant children and adolescents tend to be less physically active than their non-migrant peers. However, current research is limited as it does not sufficiently consider different domains of physical activity. Using a representative dataset, the present study examines the patterns of sports participation and other domains of physical activity among migrant and non-migrant children and adolescents residing in Germany.

**Methods:**

Nationwide data from the Motorik-Modul (MoMo) Study is used. Five different domains of physical activity participation (sports clubs, outside of sports clubs, extra-curricular physical activity, physical activity, outdoor play and active commuting to school) were compared between children and adolescents with no, one-sided and two-sided migration background using logistic regression adjusted for demographic factors. Interaction terms were included in order to examine whether difference between the three groups differ by age and gender.

**Results:**

Information on n = 3,323 children and adolescents was available. As compared to non-migrants, children and adolescents with a two-sided migration background had a 40% (adjusted odds ratio [aOR] = 0.60, 95%-CI: 0.44–0.81), those with a one-sided migration background a 26% (aOR = 0.74, 95%-CI: 0.55-<1.00) lower chance of participating in sport club activities. In contrast, children and adolescents with a two-sided migration background were at 65% higher chance of participating in extra-curricular physical activity than non-migrants (OR = 1.65, 95%-CI: 1.15–2.36).

**Conclusion:**

The study shows that differences in levels of physical activity between migrant and non-migrant children and adolescents are less pronounced than previous research has suggested. In particular, it reveals that migrants are only disadvantaged regarding participation in sports clubs whereas they fare better with respect to extra-curricular physical activity. Interventions should therefore address barriers migrant children and adolescents encounter in the access to sport clubs while maintaining their high level of extra-curricular physical activity.

## Introduction

Large proportions of the populations in many European countries are individuals who themselves or whose parents immigrated from another country [1]. In health research, they are usually referred to as migrants, immigrants or people with a migration background. In Germany, about 19.3 million individuals with German and non-German nationality, or about one quarter of the population, are migrants [2]. This population group differs from the majority population in terms of health status, health behavior and the utilization of the health care system: Amongst others, this becomes evident in a higher prevalence and incidence of certain chronic diseases, in different mortality patterns and in the underutilization of prevention and health promotion [3–7]. These inequalities result from a lower socioeconomic status, from barriers that migrants encounter in the health care system as well as from health care needs and expectations which are not sufficiently accounted for by health care providers [8, 9].

Health inequalities become not only evident among migrants of adult age but also among children and adolescents. Aside from a higher prevalence of mental disorders, a lower utilization of primary and secondary prevention and poorer oral health, migrant children and adolescents have been reported to have a higher risk of overweight and obesity [10, 11]. Compared to non-migrant children and adolescents residing in Germany, the prevalence of overweight and obesity is considerably higher for children and adolescents whose both parents are migrants themselves (commonly referred to as “two-sided migration background”) (19.5% and 8.8% vs. 14.1% and 5.9%, respectively). The prevalence among children and adolescents who only have one foreign-born parent (“one-sided migration background”) is similar to the prevalence among non-migrants.

An important determinant of overweight and obesity is a sedentary life style and poor physical activity [12]. In general, physical activity and sports are essential protective factors for many non-communicable diseases [13]. Physical activity has been defined as any bodily movement produced by skeletal muscles that requires energy [14]. In children and adolescents, this includes physical activities in daily life that can be categorized in different domains like physical education in schools, extra-curricular sports, club sports, unorganized leisure-activities, outdoor play and active commuting to school. Taking these different domains into account is necessary for the development and implementation of behavior- and context-specific physical activity promotion programs in general [15] and for migrant youth in particular [16].

Previous research examining physical activity among migrants and non-migrants in German-speaking countries is scarce: Migrant children and adolescents tend to be less physically active than their non-migrant counterparts [17, 18]. Analyses based on representative population data from Germany show that the percentage of children and adolescents who engage in regular sports activities in organized clubs are considerably lower among migrants than non-migrants, which is particularly true for girls [19–22]: While migrant adolescent girls were less likely to participate in sports in organized clubs than non-migrant adolescent girls [20], they were much more likely to walk to school [21]. In other European countries, migrant children have also been found to be more often active commuters to school than native children [23, 24]. However, they seem to have lower rates of sports clubs participation [17, 25, 26]. Thus, differences in physical activity and sports participation between migrant and non-migrant children and adolescents seem to vary across different domains. To the best of our knowledge, no comprehensive nationwide research of physical activity and sports participation among migrant and non-migrant children and adolescents has been conducted which takes the role of these domains into account and which, in addition, considers differences between children and adolescents with a one-sided migration background and a two-sided migration background, respectively. Insights into these aspects are necessary in order to inform the development of goal-oriented and targeted interventions promoting physical activity in this population group.

Using representative, population-based data for Germany, the present study examines the patterns of sports participation and other domains of physical activity among migrant and non-migrant children and adolescents residing in Germany based on domain-specific physical activity measures. To further assist in devising appropriate measures of prevention and health promotion, the study also examines whether differences in physical activity between migrants and non-migrants are moderated by gender and age.

## Methods

### Study design

The present study is based on data from the Motorik-Modul Study (MoMo Study). The MoMo Study is a nationwide longitudinal study on motor performance, physical activity, and health in children, adolescents and young adults living in Germany [27]. It is an in-depth study of the German Health and Examination Survey for Children and Adolescents (KiGGS) [28, 29], which aims to collect nationwide representative data on health status in youth and to continuously monitor health, health behavior and health development as well as health risks on a population level. For the baseline KiGGS and MoMo studies, which were conducted between 2003 and 2006, a nationwide, stratified, multi-stage probability sample with three sampling levels was drawn to ensure a diverse sample of children and adolescents in Germany [30]. In a first step, 167 primary sampling units were selected from an inventory of German communities stratified according to the so-called BIK classification system that measures the level of urbanization and the geographic distribution of the population [28]. The probability of any community being picked was proportional to the number of inhabitants younger than 18 years. Second, an age-stratified sample of 28,400 randomly selected children and adolescents was drawn from the official registers of local residents for the KiGGS study. Third, a subsample of the KiGGS study, aged 4 to 17 years, was randomly assigned to the MoMo Study. 17,641 children and adolescents participated in the KiGGS Baseline study and 12,368 participated at the second measurement point (KiGGS Wave 1 study) [31]. For the MoMo Wave 1 study, 6,076 out of the 12,368 KiGGS participants were randomly sampled. Of these, 3,994 (65.7%) finally participated in the survey which took place from 2009–2012. The non-response rate among migrants was higher than among non-migrants. Differences between responders and non-responders could also be observed with respect to socioeconomic status. In both cases, however, differences were not significant across all age groups. In the present analyses, we use data from respondents who participated in both the KiGGS and MoMo Wave 1 surveys and include children and adolescents attending primary or secondary schools and being between 6 years (age of school enrolment in Germany) and 17 years of age, totaling 3,323 individuals.

The KiGGS and the MoMo studies were approved by the Charité/Universitätsmedizin Berlin ethics committee and the Federal Office for the Protection of Data and were conducted according to the Declaration of Helsinki [32]. All participants of the MoMo Study gave their written consent to participate and were informed in detail about the study and data management by the Robert Koch Institute. Parents gave their written consent for minors and the presence of a legal guardian was mandatory for participants under the age of 15.

### Data collection

The data on socio-demographics (socioeconomic status, migration background) was obtained in the KiGGS Wave 1 survey by means of telephone-based interviews. Both parents of children and adolescents up to age 17 as well as their children (from age 11) were interviewed. Invitation letters were provided in German and if necessary in other languages (Arabic, English, Russian, Serbo-Croatian, Turkish, and Vietnamese). The survey was administered by a German-language interviewer. However, participants with limited German-language proficiency were also able to receive a paper-pencil questionnaire in languages other than German and to fill-in response themselves [31].

In the MoMo Study data on physical activity was collected at central locations at the aforementioned 167 stratified sample points in Germany which were close to the participants’ homes. In order to avoid systematic bias in the study results by regional or seasonal trends, the sequence of sample points visited for data collection was laid down beforehand in a random route planning. After being approached by an information letter and providing written informed consent, parents and their children were examined in the presence of a qualified interviewer on site [27]. Children answered a questionnaire on their physical activity behaviors (up to the age of 11 they did so with the help of their parents). The survey was conducted in German language.

Participation in both surveys was voluntary and anonymous and participants were informed about data security regulations prior to the investigation.

### Measures

#### Migration background

Migrant status was operationalized according to the definition of the Federal Statistical Office in Germany which uses the term “migration background”. Children and adolescents were considered to be migrants if they were non-German nationals or if they themselves or their parents have immigrated to Germany [33]. Following other studies in the field [34, 35], we distinguished between those who have two foreign-born parents (‘migrants with two-sided migration background’), and those who only have one foreign-born parent (‘migrants with one-sided migration background’).

#### Physical activity

We used five different outcomes of self-reported habitual physical activity as assessed by the MoMo Physical Activity Questionnaire (MoMo-PAQ) which is available elsewhere [36, 37]. The MoMo-PAQ consists of 28 items and measures the frequency, duration and intensity of physical activities in a normal week without a defined reference period to capture habitual physical activity in different domains (physical activity in sports clubs, leisure-time physical activity outside of sports clubs, extra-curricular physical activity, outdoor play, active commuting to school). The MoMo-PAQ has been pilot tested in a sample of 196 school-aged children and adolescents and data obtained with the MoMo-PAQ were shown to have acceptable validity and reliability (test-retest reliability: *ICC* = 0.68) [36]. The different domains of physical activity are each assessed by a different number of items.

Up to four different sports club activities were assessed by four items each: type of sports club activity (such as soccer or tennis), duration of each activity (minutes per session) and its frequency (times per week and months per year). These items were combined into an index reflecting minutes per week spent with sports club activities [37]. For the present study, we combined this information into the single dichotomous variable physical activity in sports clubs (no, ≥1 minutes/week).

Extra-curricular physical activity was assessed with respect to type of activity and duration (minutes per week). For the analysis, we constructed the dichotomous variable extra-curricular physical activity (no, ≥1 minutes/week).

Additionally, up to three unorganized, leisure-time sports activities taking part outside of sports clubs were assessed in the MoMo Study by means of three items each: type, duration (minutes per week) and frequency per year (in months). We combined these variables into a single outcome variable physical activity outside of sports clubs (no, ≥1 minutes/week).

Unorganized outdoor play was assessed by means of an item on days per week in which the child/adolescent plays outside (“How often do you normally play outside during a week, for example: playing tag, skipping rope or going to the swimming pool”) and an item assessing the minutes spent on average during one of those days. For purpose of analysis, we constructed the dichotomous variable outdoor play (0–3 days/week, ≥4 days/week) based on this information.

Active commuting to school was assessed by a question about how the children and adolescents commute to school most of the time [21]. In the analysis, we distinguished between commuting by car, train, bus or other non-active modes of transportation (“non-active commuting to school”) vs. commuting by “by foot or by bike, pedal-scooter or other active modes of commuting (“active commuting to school”).

#### Confounding factors

Individual-level socioeconomic status (SES) was assessed separately for both parents and included items on education, professional status, and the total household income [38]. All three SES domains were scored on a scale from 1 to 7 and combined into a composite score (range: 3–21), which was subsequently categorized into low (3–8), medium (9–14) and high (15–21) socioeconomic status according to published guidelines [39]. Adolescents with separated parents were assigned the socioeconomic status of the parent they lived with. The type of residential area was defined according to the number of residents living in the participant’s hometown, distinguishing between rural areas (<5,000 residents), small towns (5,000–19,999 residents), medium-sized towns (20,000–99,999 residents) and cities (>99,999 residents) [40]. Additionally, we considered whether respondents lived in the Western or Eastern region (incl. Berlin) of Germany.

### Statistical analysis

Sociodemographic characteristics were analyzed using descriptive statistics, χ^2^- and analysis of variance were appropriate. To account for confounding we conducted a logistic regression analysis for each of the five dichotomous outcomes, reporting adjusted odds ratios (aOR) and 95%-confidence intervals. To examine whether the association between migration background and the outcomes are moderated by gender and age, respective interaction effects were included into the models. They were assessed and illustrated based on average marginal effects to account for unobserved heterogeneity [41]. The analyses were adjusted for socioeconomic status, type of residential area, and region in Germany. A significance level of 0.05 was set as a threshold to determine statistical significance. All analyses were conducted with Stata version 13 [42].

## Results

Information on n = 3,323 children and adolescents was available. Of these, n = 210 (6.3%) had two foreign-born parents (“two-sided migration background”) and n = 230 (6.9%) had only one foreign-born parent (“one-sided migration background”). Descriptive statistics revealed that the three groups of study participants differed from each other in terms of some demographic and socioeconomic factors. Children and adolescents with a two-sided migration background tended to be slightly older (mean age = 12.2) than their non-migrant counterparts (mean age = 11.8) and than those with a one-sided migration background (mean age = 11.4). They were also socioeconomically more disadvantaged, with almost one quarter (24.3%) having a low socioeconomic status as compared to 6.7% and 5.7% among non-migrants and children and adolescents with a one-sided migration background. Differences also existed with respect to the location of residence with non-migrants living more often in rural and small-town areas (Table 1).

**Table 1:** Description of the study sample (data from the Motorik-Module Study [MoMo] Study; n = 3,323).

	Migration background			p-value
	No (n = 2,883)	One-sided (n = 230)	Two-sided (n = 210)	
**Gender**				0.56
Male	1,399 (48.5%)	112 (48.7%)	110 (52.4%)	
Female	1,484 (51.5%)	118 (51.3%)	100 (47.6%)	
**Age** (in years), mean (SD)	11.8 (3.1)	11.4 (3.2)	12.2 (3.2)	0.02
**Socio-economic status**				<0.01
Low	194 (6.7%)	13 (5.7%)	51 (24.3%)	
Medium	1,898 (65.8%)	147 (63.9%)	125 (59.5%)	
High	791 (27.4%)	70 (30.4%)	34 (16.2%)	
**Type of school**				0.354
Primary	1,034 (35.9%)	91 (39.6%)	69 (32.9%)	
Secondary	1,849 (64.1%)	139 (60.4%)	141 (67.1%)	
**Region in Germany**				<0.501
West	1,855 (64.3%)	196 (85.2%)	190 (90.5%)	
East (incl. Berlin)	1,028 (35.7%)	34 (14.8%)	20 (9.5%)	
**Type of residential area**				<0.015
Rural area	740 (25.7%)	41 (17.8%)	9 (4.3%)	
Small town	945 (32.8%)	64 (27.8%)	52 (24.8%)	
Medium-sized towns	787 (27.3%)	67 (29.1%)	86 (41.0%)	
Cities	411 (14.3%)	58 (25.2%)	63 (30.0%)	
**Participation in sports clubs**				<0.501
Yes	1,905 (66.7%)	148 (64.6%)	114 (56.2%)	
**Participation in extra-curricular physical activity**				0.259
Yes	550 (20.0%)	40 (17.9%)	48 (23.9%)	
**Participation in physical activity outside of sports clubs**				0.575
Yes	1,344 (48.2%)	106 (47.1%)	94 (45.6%)	
**Regular outdoor play (≥4 days/week)**				0.501
Yes	2,250 (78.7%)	174 (75.7%)	146 (70.2%)	
**Active commuting to school**				<05.01
Yes	1,370 (47.8%)	120 (52.9%)	124 (59.0%)	

*Note*. SD: standard deviation.

In terms of the five outcomes of interest, children and adolescents with a two-sided migration background as compared to non-migrants and children and adolescents with a one-sided migration background engaged less frequently in sport clubs (56.2% vs. 66.7% and 64.6%, respectively) as well as in outdoor play (70.2% vs. 78.7% and 75.7%, respectively). However, they more often commuted to school by foot or another active mode (59.0% vs. 47.8% and 52.9%, respectively). No differences between the three groups existed with respect to participation in extra-curricular physical activity and physical activity outside of sport clubs (Table 1).

Table 2 shows the results of the multivariable logistic main effects model, separately for each of the five outcomes. Significant differences between the three groups of children and adolescents only become evident in terms of physical activity related to sport clubs. As compared to non-migrants, children and adolescents with a two-sided migration background had a 40% (OR = 0.60, 95%-CI: 0.44–0.81), children and adolescents with a one-sided migration background a 26% (OR = 0.74, 95%-CI: 0.55-<1.00) lower chance of participating in sport club activities.

**Table 2:** Results of the multivariable logistic regression analysis of five domains of physical activity (adjusted odds ratios [aOR] and 95%-confidence intervals [95%-CI]; data from the Motorik-Module Study [MoMo] Study; no interaction effects included).

	Domain of physical activity									
	Sports clubs (n = 3,286)		Extra-curricular physical activity (n = 3,172)		Outside of sports clubs (n = 3,219)		Outdoor play (n = 3,297)		Active commuting to school (n = 3,305)	
	aOR	95%-CI	aOR	95%-CI	aOR	95%-CI	aOR	95%-CI	aOR	95%-CI
**Migration background**										
No	1		1		1		1		1	
One-sided	0.74	[0.55-<1.00]	0.99	[0.68–1.42]	0.95	[0.72–1.25]	0.73	[0.49–1.07]	1.02	[0.77–1.36]
Two-sided	0.60	[0.44–0.81]	1.65	[1.15–2.36]	0.93	[0.69–1.25]	0.79	[0.53–1.17]	1.19	[0.88–1.61]
**Age** (in years)	0.93	[0.90–0.97]	0.92	[0.88–0.96]	1.07	[1.04–1.11]	0.60	[0.57–0.63]	0.97	[0.94–1.01]
**Gender**										
Male	1		1		1		1		1	
Female	0.58	[0.50–0.68]	0.82	[0.69–0.98]	0.87	[0.76–1.00]	0.57	[0.47–0.70]	1.05	[0.91–1.21]
**Socio-economic status**										
Low	1		1		1		1		1	
Medium	1.86	[1.42–2.45]	0.95	[0.68–1.33]	1.22	[0.93–1.60]	1.22	[0.86–1.73]	1.14	[0.86–1.50]
High	3.10	[2.29–4.20]	0.93	[0.65–1.33]	1.50	[1.12–2.01]	1.13	[0.77–1.65]	1.31	[0.98–1.77]
**Type of school**										
Primary	1		1		1		1		1	
Secondary	1.30	[1.02–1.64]	0.84	[0.63–1.12]	0.86	[0.69–1.07]	0.77	[0.54–1.11]	0.70	[0.56–0.88]
**Region in Germany**										
West	1		1		1		1		1	
East (incl. Berlin)	0.45	[0.38–0.52]	2.04	[1.70–2.46]	0.84	[0.72–0.98]	0.88	[0.71–1.09]	0.71	[0.61–0.83]
**Type of residential area**										
Rural area	1		1		1		1		1	
Small town	1.09	[0.89–1.34]	0.86	[0.68–1.09]	0.90	[0.74–1.08]	1.11	[0.84–1.45]	2.30	[1.88–2.81]
Medium-sized towns	0.97	[0.78–1.19]	0.88	[0.68–1.12]	0.79	[0.65–0.96]	0.79	[0.60–1.04]	4.09	[3.32–5.04]
Cities	0.87	[0.68–1.11]	0.83	[0.62–1.11]	0.83	[0.66–1.05]	0.59	[0.43–0.81]	2.69	[2.12–3.42]

*Note*. aOR: adjusted odds ratios; 95%-CI: 95%-confidence interval

Unlike in the descriptive analysis, the adjusted analysis revealed that children and adolescents with a two-sided migration background were at 65% higher chance of participating in extra-curricular sports (OR = 1.65, 95%-CI: 1.15–2.36). No significant difference between the three groups existed with respect to the other three physical activity domains.

Aside from migration background, significant associations could also be observed with regard to the different covariates studied, although the overall picture was inconstant.

In the two physical activity outcomes associated with migration background, no significant interaction effects could be observed between migration background and age or between migration background and gender.

## Discussion

The aim of the current study was to examine the patterns of physical activity and sports participation among migrant and non-migrant children and adolescents residing in Germany based on domain-specific physical activity measures. The domain-specific analyses of physical activity participation in 3,323 children and adolescents from Germany revealed that differences in physical activity participation of migrant and non-migrant children and adolescents varied with respect to physical activity domains. The consideration of different domains of physical activity is warranted because correlates of physical activity are claimed to be behaviour- and context-specific [15], and physical activity in different domains has different effects on health outcomes [43]. Thus, it is necessary to distinguish between physical activity domains to identify potential disadvantages between migrant and non-migrant children and adolescents and to further investigate supporting factors and barriers of physical activity in different population groups. These factors are important starting points for the development of tailored interventions for the promotion of physical activity and healthy active living.

In the current study, children and adolescents with a migration background were only disadvantaged with respect to physical activity undertaken in sports clubs, but not in relation to any of the other domains. Children and adolescents with a two-sided migration background were even more likely to take part in extra-curricular physical activity than their non-migrant peers. While descriptive results also revealed a lower percentage playing outside and a higher percentage of active school commuters among migrant children and adolescents as compared to non-migrants, these relationships were not significant in the multivariable models. This indicates that other (confounding) variables need to be taken into account as explanatory factors [44]. For example, the type of residential area (ranging from rural to city) is strongly related to active commuting to school as the average distances to school are longer in rural areas in comparison to medium-sized towns or cities [21]. Since migrant children and adolescents are more likely to live in medium-sized towns or cities than their non-migrant counterparts, it is suggested that they are more often active commuters.

Moderating effects on the relationship of migration status and physical activity based on gender and age have not been found. This contradicts previous studies which described disadvantages in physical activity participation particularly in migrant girls (and simultaneously higher proportions of sport club participation among migrant as compared to non-migrant boys) [20, 45]. This highlights the role of intersectionality and calls for the identification of intersectional differences in physical activity [46], because instead of differences between migrant and non-migrant youth there might be disadvantages in specific sub-groups of migrants that for instance concern a specific age or gender.

It has been shown that migrant children and adolescents were less likely to participate in physical activity in sports clubs regardless of age, gender and socioeconomic profiles. Previous studies conducted in Germany also confirmed that migrant adolescents were underprivileged with regard to sports clubs participation and postulated that aspects like cultural differences, socioeconomic disadvantages, intergenerational inheritance of sports engagement, and ethnic discrimination are responsible for generating inequalities [20, 45]. However, these studies strongly stressed that disadvantages in physical activity participation in sports clubs vary by gender. The differences in sports participation between migrants and non-migrants were much larger among adolescent girls than boys [45]. Consequently, sports clubs seemed to be less accessible especially for adolescent migrant girls. This finding, however, has not been confirmed in the current study. Presumably, integration programs developed by the German Olympic Sports Confederation such as the “Integration through Sport” program (www.integration-durch-sport.de) helped to make sports clubs better accessible for migrant children and adolescents.

Despite disadvantages with respect to physical activity in sports clubs, extra-curricular physical activity was found to be more prevalent in children and adolescents with a two-sided migration background than among non-migrants. This finding confirms the results of a regional study from Germany conducted in 1992 [47] and the results of the German SPRINT-study conducted in 2004 [48]. Furthermore, in Germany, school-age migrant children engage in extra-curricular school programs generally more often [49], potentially compensating a limited access to sport clubs and thus resulting in a higher proportion of participation. Extra-curricular physical activity programs are more accessible to a broad range of children and adolescents because they are usually free of charge and within easy reach as they often take place in the school area where the school children are present after regular school lessons. The results, therefore, stress that extra-curricular physical activity is a domain where migrants could be successfully reached to promote physical activity. Still, existing barriers that limit migrant children and adolescent in their access to regular sport club activities need to be addressed in order to promote their equal access to social participation.

To the best of our knowledge, no studies from Germany are available that compare migrant and non-migrant children with respect to the frequency of outdoor play. A study conducted in the Netherlands showed that native Dutch children were more likely to play outdoors >1 hour per day than ethnic minority children [50]. However, a study from Denmark showed that 6- to 7-year-old migrant children were more often playing outdoors than non-migrant children [51]. Thus, the relevance of migration status for the level of outdoor play in children is inconclusive. In the present study the significant association disappears in the multivariable analysis indicating that other factors like age and gender were more relevant. Future research on disparities in outdoor play also needs to take into account the role of contextual factors such as the availability of near-by playgrounds, which are associated with the participation in outdoor activities [52, 53].

Some other studies also found migrant youth being more frequently active commuters to school or cumulating more minutes per day in active transportation than non-migrant youth in Germany [21] and other European countries like Belgium, the Netherlands or Greece [23, 24]. However, when distinguishing between modes of active transports it appears that migrant adolescents are more likely to walk to school but less likely to cycle to school than non-migrants [21, 23]. This could be due to cultural habits or socioeconomic status as migrant children and adolescents are overrepresented in the low socioeconomic status group [33] and financially not able or used to buy a bike. Nevertheless, in the current study (in the descriptive analysis) and other studies, migrants were more often active commuters to school indicating that active commuting to school could be a relevant source of physical activity in this group.

### Strengths and limitations

Strengths of the current study are the consideration of different domains of physical activity and the distinction between children and adolescents with a one-sided and a two-sided migration background, which enabled us to draw a more detailed picture of physical activity in migrants of this age group. The large sample size and the inclusion of school-aged children with a wide age range also allowed to examine differences between migrants and non-migrants from childhood through adolescence. Additionally, the collection of data from a nationwide sample of school-aged children and adolescents from 167 communities in Germany pleads for a high degree of generalizability.

Nevertheless, some limitations of the study have to be mentioned. First, due to language and cultural barriers [54], it was difficult to ensure a high response-rate especially in migrant participants [31]. Thus, in spite of oversampling and additional measures such as the use of multilingual information material and questionnaires in the KiGGS study, the percentage of migrant participants in our study does not match with census data [2]. It is, therefore, possible that our sample is not representative for the whole population of school-aged migrant children and adolescents in Germany. In particular, it can be assumed that individuals with limited German-language proficiency are underrepresented. Future surveys, therefore, should draw on multilingual interviewers to collect data in this population group. Second, we were not able to distinguish between different ethnic backgrounds. There might be differences between children and adolescents from different ethnic origins as shown by other studies in the field [55–58]. Third, all data is based on self- or proxy-reports and is thus prone to bias, including recall bias and social desirability. Measuring physical activity by objective methods like accelerometry has been shown to be more accurate [59]. However, the objective measurement of physical activity in different domains can be challenging and requires complex strategies which can hardly be applied in large samples.

## Conclusion

The current study shows that disparities in physical activity between migrant and non-migrant children and adolescents may vary considerably by different domains of activity and seem less pronounced than previous research has suggested. A lower proportion of participation among migrants became only visible with respect to the participation in sport clubs and seems to be compensated by a higher participation with respect to extra-curricular sports. Whereas barriers migrant children and adolescents face in the access to sport clubs need to be addressed by appropriate interventions, their higher level of extra-curricular sports activities needs to be maintained. Further insights into their motivation to engage in this domain of activity could also inform measure which aim to increase extra-curricular sports activities in non-migrants.
